# The variability of giraffe skull morphology

**DOI:** 10.1111/joa.70157

**Published:** 2026-05-01

**Authors:** Nikolaos Kargopoulos, Jesús Marugán‐Lobón, Anusuya Chinsamy‐Turan, Bernard R. Agwanda, Michael Butler Brown, Stephanie Fennessy, Sara Ferguson, Rigardt Hoffman, Fredrick Lala, Arthur Muneza, Ogeto Mwebi, Moses Otiende, Alice Petzold, Sven Winter, Abdoul Razack Moussa Zabeirou, Julian Fennessy

**Affiliations:** ^1^ Department of Biological Science University of Cape Town Cape Town South Africa; ^2^ Giraffe Conservation Foundation Windhoek Namibia; ^3^ Unidad de Paleontología, Department of Biology Universidad Autónoma de Madrid Madrid Spain; ^4^ CIPb‐UAM, Center for the Integration in Paleobiology Universidad Autónoma de Madrid Madrid Spain; ^5^ National Museums of Kenya Nairobi Kenya; ^6^ UMP‐Oppenheimer Chair in Biodiversity Conservation, Faculty of Agriculture and Natural Sciences University of Mpumalanga Mbombela South Africa; ^7^ Wildlife Research and Training Institute Naivasha Kenya; ^8^ Institute of Biochemistry and Biology University of Potsdam Potsdam Germany; ^9^ Museum für Naturkunde – Leibnitz Institute for Evolution and Biodiversity Science Berlin Germany; ^10^ Senckenberg Biodiversity and Climate Research Centre Frankfurt am Main Germany; ^11^ Faculty of Science and Technology University of the Faroe Islands Tórshavn Faroe Islands; ^12^ Faculty of Tropical AgriSciences Czech University of Life Sciences Prague Prague Czech Republic; ^13^ Sahara Conservation Niamev Niger; ^14^ School of Biology and Environmental Science University College Dublin Dublin Ireland

**Keywords:** allometry, evolution, *Giraffa*, integration, ontogeny, taxonomy

## Abstract

Giraffe (*Giraffa* spp.) are among the most unique and charismatic extant animals, largely due to their distinct morphology. The evolutionary and developmental origin of ossicones is a key factor in giraffid biology, yet other features building their characteristic head physiognomy remain poorly studied. Here, we analysed the cranial diversity of extant *Giraffa* using a continent‐wide sample with a balanced sex ratio and ontogenetic growth series, using shape analysis (3D geometric morphometrics). Detailed comparisons showed significant differences in cranial morphology across the four *Giraffa* species, and in some cases also between subspecies of the same species. The analyses revealed that females reach adult cranial form at an earlier age (around 4 years old), while males continue growing until the eighth year, aligning with the onset of sexual maturity for each of the sexes. Multivariate methods unambiguously prompt significant covariation between skull parts. Such high covariation patterns indicate that *Giraffa* skull morphology largely evolves as a whole (i.e., morphologically integrated), even though parts of it (such as the ossicones) develop in different magnitudes. Considering their role in territorial and mating behaviours, we suggest that ossicone morphology influences the parapatric evolution of *Giraffa* through sexual selection. Palaeontological evidence shows that development of larger ossicones is a relatively recent trait for members of the genus *Giraffa*, with the cranial morphology of the southern giraffe (*Giraffa giraffa*) being the closest to that of the ancestral forms, possibly through plesiomorphy or paedomorphosis.

## Graphical abstract

What does giraffe skull shape tell us about their development and evolution?

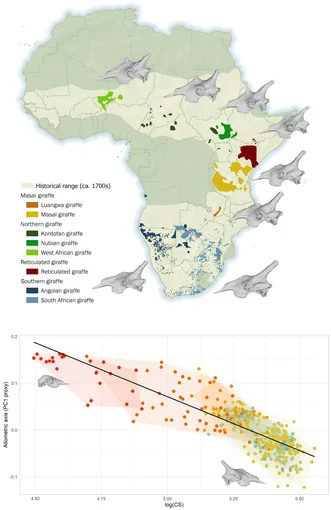

## 1 INTRODUCTION

The giraffe (*Giraffa* spp.) is as iconic as it is enigmatic – an animal whose origins, striking overall physique, and recent diversification have been difficult to disentangle for over 200 years (e.g., Mitchell, 2009; Muneza et al., 2025; Seymour, 2001; Shorrocks, 2016). Such knowledge is crucial not only for advancing science but also for conservation, given that international assessments of conservation status are typically conducted at the species level (e.g., Brown et al., 2023; Costello et al., 2015; Mace, 2004; Marneweck et al., 2025; Morrison III et al., 2009; Muller et al., 2018; Muneza et al., 2025; Schmidt et al., 2023; Vogel Ely et al., 2017). Recent breakthroughs in taxonomic assessment stem from new technological advances, including molecular biology and digital shape analytical tools (e.g., geometric morphometrics, GM). Such approaches have provided fresh insights into the existence of four giraffe species and eight subspecies, identifiable genetically (Bertola et al., 2024; Bock et al., 2014; Brown et al., 2007; Coimbra et al., 2021; Fennessy et al., 2016, 2017; Hassanin et al., 2007; Winter et al., 2018) but also by differences in cranial traits that contribute to skull shape (Kargopoulos et al., 2024; Seymour, 2001) (Figure 1). Moreover, whole‐genome assessments have demonstrated the uniqueness of *Giraffa* among ungulates, revealing that historical phylogenetic difficulties in classifying them arise from their largely reticulate evolution (Bertola et al., 2024; Coimbra et al., 2021; Fennessy et al., 2016; Winter et al., 2018). Specifically, divergence with gene flow has played a significant role in their evolutionary history (Bertola et al., 2024).

**FIGURE 1:**
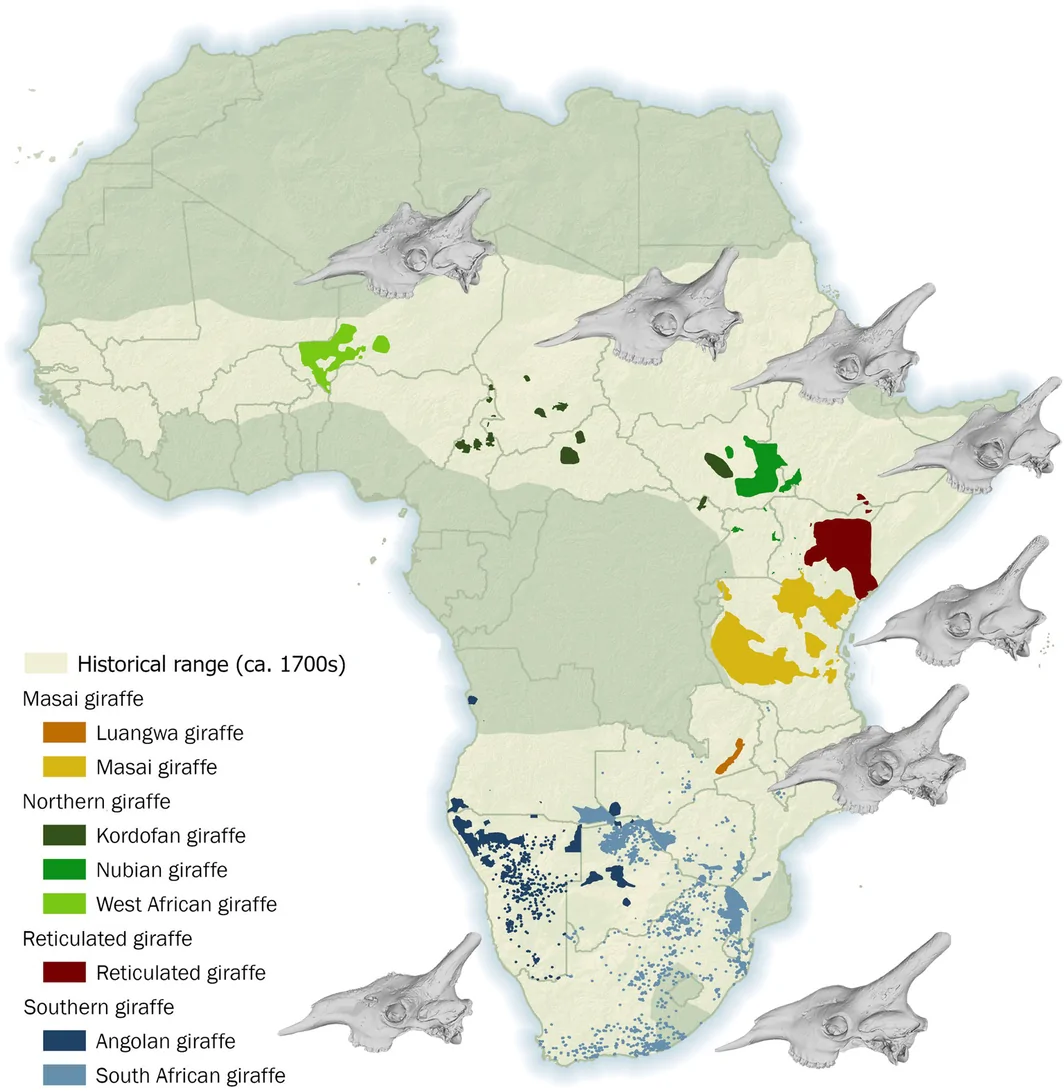
Map showing the distribution of *Giraffa* species and subspecies, as well as representative male skulls from each one of them.

The *Giraffa* skull, as in all mammals, is anatomically complex and can be divided into distinct functional parts, such as the face, the braincase, the occiput, and the cranial ornaments, with each part characterized by specific adaptations that aid in the completion of specific tasks (e.g., Cheverud, 1982). Skull physiognomy, in effect, is the most striking visual feature of giraffe, alongside their elongated legs, neck, and distinctive pelage. Notably, the *Giraffa* skull is large but also bears three ossicones as well as expanded areas of secondary ossification (nuchal crest, orbits, nasals, etc.), along with a prominent dome‐shaped frontal sinus that bulges beneath the bases of the ossicones in adults (Murie, 1872; Ridewood, 1904; Spinage, 1968, 1970, 1993; Thomas, 1901). While the most notable differences between species or sexes are observed in the ossicones (Kargopoulos et al., 2024), other features concerning the rostrum, the palates, the orbits, and the nuchal crest also show variation. The dorsal part of the skull includes the ossicones and the nuchal crest that can be used as a means of display and aggression. In males, secondary ossification including ossification centres on the ossicones, nuchal crest, orbits, rostrum, and surfaces between them, covers most of the dorsal part of their skulls to strengthen this surface and enable greater force during male–male fights and to appear more intimidating (e.g., Dagg, 1971; Simmons & Altwegg, 2010; Singer & Boné, 1960; Spinage, 1968, 1970, 1993). Cranial ornaments in ungulates, such as antlers, horns, and ossicones, play crucial roles in intraspecific competition, mate attraction, and species recognition (Bubenik & Bubenik, 1990; and references therein). These structures function as weapons in male–male combat for dominance, honest signals of genetic fitness in sexual selection, and are visual markers for differentiating species or individuals. The evolution of these traits is theoretically driven by the pressures of sexual and natural selection, balancing reproductive benefits with the energetic and survival costs of maintaining them (Futuyma & Kirkpatrick, 2022). Under evolutionary theory, such traits are expected to evolve independently in response to the specific selection pressures acting upon them, that is, as evolutionary modules (Wagner et al., 2007). Interestingly, the cranial morphology of *Giraffa* has never been thoroughly assessed in this context of integration and phylogeny.

Developmentally, the skull follows growth‐based pathways (allometry) that shape it as an integrated whole. Yet, distinct evolutionary (selective regimes) or developmental (morphogenesis) processes may result in enhanced integration or independence of its parts, a phenomenon known as modularity (Chernoff & Magwene, 1999; Klingenberg, 2009; Olson & Miller, 1958; Wagner et al., 2007). In such a scenario, the modularity of cranial ornaments would be significantly higher than that of the rest of the skull (Gerstenhaber & Knapp, 2022; Haber, 2015). To date, however, the modular organization, ontogenetic variation, and evolutionary significance of *Giraffa* cranial morphology have not been explicitly investigated. Here, we build upon the 3D geometric morphometric data presented by Kargopoulos et al. (2024) to explore this gap. Specifically, we aim to: (1) identify finer scale morphological differences between taxonomic groups through targeted comparisons, (2) analyse how skull morphology changes throughout ontogeny and assess the role of allometry in shaping developmental trajectories, (3) determine whether skull shape changes occur in an integrated manner across species or whether cranial ornaments act as independent modules, as theoretically predicted, and (4) interpret these patterns within a broader ecological and evolutionary context to better understand the drivers and implications of *Giraffa* cranial variability.

## 2 MATERIALS AND METHODS

### 2.1 Materials

We studied 515 extant *Giraffa* skulls, a summary of which is presented in Table 1. The skulls were scanned from museum collections (*n* = 363, 70.5%), wild individuals obtained from parks and reserves throughout Africa (*n* = 70, 13.6%), and specimens provided by taxidermists originating from known parks and reserves in southern Africa (*n* = 82, 15.9%). The limited geographical overlap between extant *Giraffa* subspecies enabled us to accurately identify the taxon of most specimens based on its provenance. The specimens identified as *Giraffa* sp., *Giraffa camelopardalis* subsp., and *Giraffa giraffa* subsp. do not have adequate provenance data to attribute them to a more specific taxon.

**TABLE 1:** Summary of the material studied herein.

Subspecies	Common name	M	F	X	A–D	E–I	Total
*Giraffa cam*. *antiquorum*	Kordofan	21	17	0	8	30	38
*Giraffa cam*. *camelopardalis*	Nubian	41	17	0	8	46	54
*Giraffa cam*. *peralta*	West African	5	4	2	1	10	11
*Giraffa cam*. subsp.	–	7	5	0	4	8	12
*Giraffa ret*. *reticulata*	Reticulated	20	12	2	8	26	34
*Giraffa tip*. *tippelskirchi*	Masai	58	43	7	31	77	108
*Giraffa tip*. *thornicrofti*	Luangwa	17	3	1	4	17	21
*Giraffa gir*. *angolensis*	Angolan	31	12	0	12	31	43
*Giraffa gir*. *giraffa*	South African	90	26	5	34	87	121
*Giraffa gir*. subsp.	–	9	6	0	2	13	15
*Giraffa* sp.	–	32	21	5	18	40	58

*Note*: M: Males, F: Females, and X: Unknown sex; A–D: Juvenile and subadult age classes A–D, E–I: Adult age classes E–I.

The abbreviations of the visited museums, National Parks, and taxidermy collections visited during the course of this study are as follows:

Museums: AMNH – American Museum of Natural History, New York (USA); AMPG – Athens Museum of Palaeontology and Geology (Greece); DNMNH – Ditsong National Museum of Natural History, Pretoria (South Africa); FMNH – Field Museum of Natural History, Chicago (USA); HNHM – Hungarian Natural History Museum, Budapest (Hungary); NCB – Naturalis Biodiversity Center, Leiden (the Netherlands); NHMUK – Natural History Museum of the United Kingdom, London (United Kingdom); NHMZ – Natural History Museum of Zimbabwe, Bulawayo (Zimbabwe); NMB – Naturhistorisches Museum Basel (Switzerland); NMK – National Museums of Kenya, Nairobi (Kenya); NMW – Naturhistorisches Museum Wien (Austria); RMCA – Royal Museum of Central Africa, Tervuren (Belgium); SAM – Iziko South African Museum, Cape Town (South Africa); SMF – Senckenberg Museum Frankfurt (Germany); SMNS – Staatliches Museum für Naturkunde Stuttgart, Stuttgart (Germany); SNM – Slovak National Museum, Bratislava (Slovakia); UCT – University of Cape Town (South Africa); USNM – United States National Museum, Smithsonian Institute, Washington DC (USA); ZFMK – Zoological Research Museum Alexander Koenig, Bonn (Germany); ZMUA – Zoological Museum, University of Athens (Greece); ZMB – Museum für Naturkunde, Berlin (Germany); ZSM – Zoologische Staatssammlung, Staatlichen Naturwissenschaftlichen Sammlungen Bayerns, Munich (Germany).
Natural parks: CHA‐ZAK – Zakouma (Chad), KEN‐AMB – Amboseli NP, Kenya; KEN‐MAM – Maasai Mara NP, Kenya; KEN‐MER – Meru, Kenya; KEN‐MGE – Mgeno Conservancy, Kenya; KEN‐RUM – Ruma NP, Kenya; KEN‐TSE – Tsavo East NP, Kenya; KEN‐TSW – Tsavo West, Kenya; NAM‐ETH – Etosha Heights Private Reserve (Namibia); NER‐KOU – Kouré Giraffe Reserve (Niger); UGA‐MUR – Murchison Falls (Uganda); ZAM‐LUA – Luangwa Valley (Zambia).
Taxidermy collections: NAM‐ING – Ingwe (Namibia), NAM‐NYA – Nyati (Namibia), NAM‐TAU – Tau (Namibia); SA‐SI – Splitting Image (South Africa); ZIM‐TCI – Trophy Consultants International (Zimbabwe).

### 2.2 Methods

The studied specimens were 3D‐ or CT‐scanned to acquire 3D models, which were then used for further analysis. Most of the specimens were scanned using an EinScan Pro HD handheld scanner; however, the specimens at ZFMK were scanned with an Artec Space Spider 3D scanner, while those at ZMB and SA‐SI were scanned with an Artec Leo 3D scanner. The skulls from NER‐KOU were CT‐scanned at Polyclinique Magori (Niamey, Niger; slice 0.75, kernel B20s) and segmented using 3D Slicer (Fedorov et al., 2012).

The determination of age classes followed the methodology of Kargopoulos et al. (2024), based on suture closure and dental wear (Hall‐Martin, 1976; see Table 2). Figures aiding the attribution to these categories can be found in the Figures S1–, S4.

**TABLE 2:** Age classes of giraffe skulls and their characteristics.

Age class	Actual age	Characteristics
A	<1	Only deciduous cheek teeth present. Interparietal suture at least partially open. Metopic and coronal sutures open
B	1–4	Deciduous and fully erupted adult cheek teeth present. M1 distal loop closed. Presence of enamel between the paracone and metacone of M1. Pulp cavities of M1 not visible. Interparietal suture at least partially open. Metopic and coronal sutures open
C	4–6	Only adult cheek teeth present. M1 distal loop closed. Presence of enamel between the paracone and metacone of M1. Pulp cavities of M1 not visible. Interparietal suture closed. Metopic and coronal sutures open.
D	6–8	Only adult cheek teeth present. M1 distal loop closed. Presence of enamel between the paracone and metacone of M1. Pulp cavities of M1 not visible. Interparietal suture closed. Metopic and coronal sutures partially closed
E	8–11	Only adult cheek teeth present. M1 distal loop closed. Presence of enamel between the paracone and metacone of M1. Pulp cavities of M1 not visible. Interparietal suture closed. Metopic and coronal sutures closed
F	11–14	Only adult cheek teeth present. M1 distal loop open. Presence of enamel between the paracone and metacone of M1. Pulp cavities of M1 not visible. Interparietal suture closed. Metopic and coronal sutures closed
G	14–17	Only adult cheek teeth present. M1 distal loop open. Presence of dentine connection between the paracone and metacone of M1. Pulp cavities of M1 not visible. Interparietal suture closed. Metopic and coronal sutures closed
H	17–20	Only adult cheek teeth present. M1 distal loop open. Complete absence of enamel between the paracone and metacone of M1. Pulp cavities of M1 not visible. Interparietal suture closed. Metopic and coronal sutures closed
I	>20	Only adult cheek teeth present. M1 distal loop open. Complete absence of enamel between the paracone and metacone of M1. Complete absence of enamel and possibly visible pulp cavities of M1. Interparietal bone not visible. Metopic and coronal sutures closed

3D landmarks were placed on the skulls according to the protocol reported by Kargopoulos et al. (2024) using Stratovan Checkpoint (Stratovan Corporation, 2024) (Figure S5). The landmark coordinates were then imported into R 4.5.1 and estimation of missing landmarks and generalized procrustes analyses (GPA) were performed in the R package *geomorph* 4.0.10 (Baken et al., 2021). The conducted analyses included multiple regressions, principal component analysis – PCA, canonical variate analysis – CVA, two‐block partial least squares – 2B‐PLS, modularity analysis, all paired with correlated robustness tests. Levene's test was used to evaluate the homogeneity of dataset's before the analyses (Brown & Forsythe, 1973), while leave‐one‐out cross‐validation (LOO) was used to evaluate the performance of the CVAs (Wong, 2015). *Morpho* 2.13 (Schlager, 2017) was used to read the landmark files and *geomorph* 4.0.10 (Baken et al., 2021) was used for the GPA together with *rgl* 1.3.24 (Murdoch & Adler, 2025). Linear analyses and ANOVA were performed with *MASS* 7.3–65 (Venables & Ripley, 2002) and *car* 3.1–3 (Fox & Weisberg, 2019) respectively. Visualization of the analyses results was made possible with *ggplot2* 3.4.4 (Wickham, 2016), *wesanderson* 0.3.7 (Ram & Wickham, 2023), *scales* 1.4.0 (Wickham et al., 2025a, 2025b), *ggrepel* 0.9.6 (Slowikowski, 2025), and *ggforce* 0.5.0 (Pedersen, 2025). Data handling was made easier with the use of *openxlsx* 4.2.8 (Schauberger & Walker, 2025), *dplyr* 1.1.4 (Wickham et al., 2025a, 2025b), *tibble* 3.3.0 (Müller & Wickham, 2025), and *biotools* 4.3 (da Silva, 2025). Phylomorphospaces were created using R *phytools* 2.5–2 (Revell, 2024) together with *ape* 5.8–1 (Paradis & Schliep, 2019) and *RRPP* 2.1.2 (Collyer & Adams, 2018), using the phylogenetic hypothesis of Coimbra et al. (2021) through Mesquite 3.81 (Maddison & Maddison, 2023). The code can be found on https://github.com/nkargopoulos/Giraffe‐Cranial‐3D‐GM. The *p*‐values of these tests are listed in Table S1. The visual expression of the shape changes represented along the axes of the analyses were obtained using skull morphing in Landmark Editor (IDAV, 2005) over specimen NMB‐12075 (*G*. *c*. *antiquorum*, female, age class G).

Some analyses were conducted focusing on specific groups; for example, only in the North group of *camelopardalis* and *reticulata* and separately in the South group of *giraffa* and *tippelskirchi*. Such separations were performed to clarify the more subtle difference between the taxonomic groups that were not so clearly seen in lumping plots.

Allometry (relationship between shape and size) is a first level of morphological integration that can be evaluated as such (i.e., ontogenetic) or between adults (static) (Klingenberg & McIntyre, 1998). In shape analysis based on Procrustes data, it can be assessed using multivariate regressions (Monteiro, 1999). To such end, our sample encompasses a late ontogeny growth series including females and males. To further assess the levels of integration and modularity (i.e., the degree of covariation between skull parts), the landmark series was divided into ad hoc selected skull partitions, which were then submitted to a two‐block partial least squares analysis (2B‐PLS). This multivariate technique is a test of pairwise inter‐partition covariance between blocks of shape data by identifying pairs of linear combinations (latent variables) that explain the maximal shared variation between them (Rohlf & Corti, 2000). Accordingly, one test compared the ventral and dorsal parts of the skull (Figure 2a), which encompassed a separation between the ornaments and the craniofacial system (i.e., the neurocranium and face). The ventral segment (Block 1) included the single points 1–7 (premaxillae), 14–34 (maxilla, infraorbital foramina, palatal foramina, palate end, optic, rotundum, zygomatic), and 35–42 (paraoccipital, ear, basisphenoid, foramen magnum), as well as the curves 6–7 (toothrows) and the ventral part of the curves 4–5 (orbits). The dorsal segment (Block 2) included the single points 8–13 (nasals), as well as the curves 1–3, 8 (sagittal, ossicones, nuchal), and the dorsal part of the curves 4–5 (orbits). Another test was performed between the neurocranium (Block 1) and the face (Block 2) (Figure 2b). The neurocranium segment (Block 1) includes the single points 29–42 (optic, rotundum, zygomatic, paraoccipital, ear, basisphenoid, foramen magnum), as well as the curves 2 and 3 and the distal part of curve 1. The face segment (Block 2) includes the single points 1–28 (premaxillae, nasals, maxilla, infraorbital foramina, palatal foramina, palate end), as well as the curves 4–8 (orbits, toothrows, medial ossicone plane) and the proximal part of curve 1.

**FIGURE 2:**
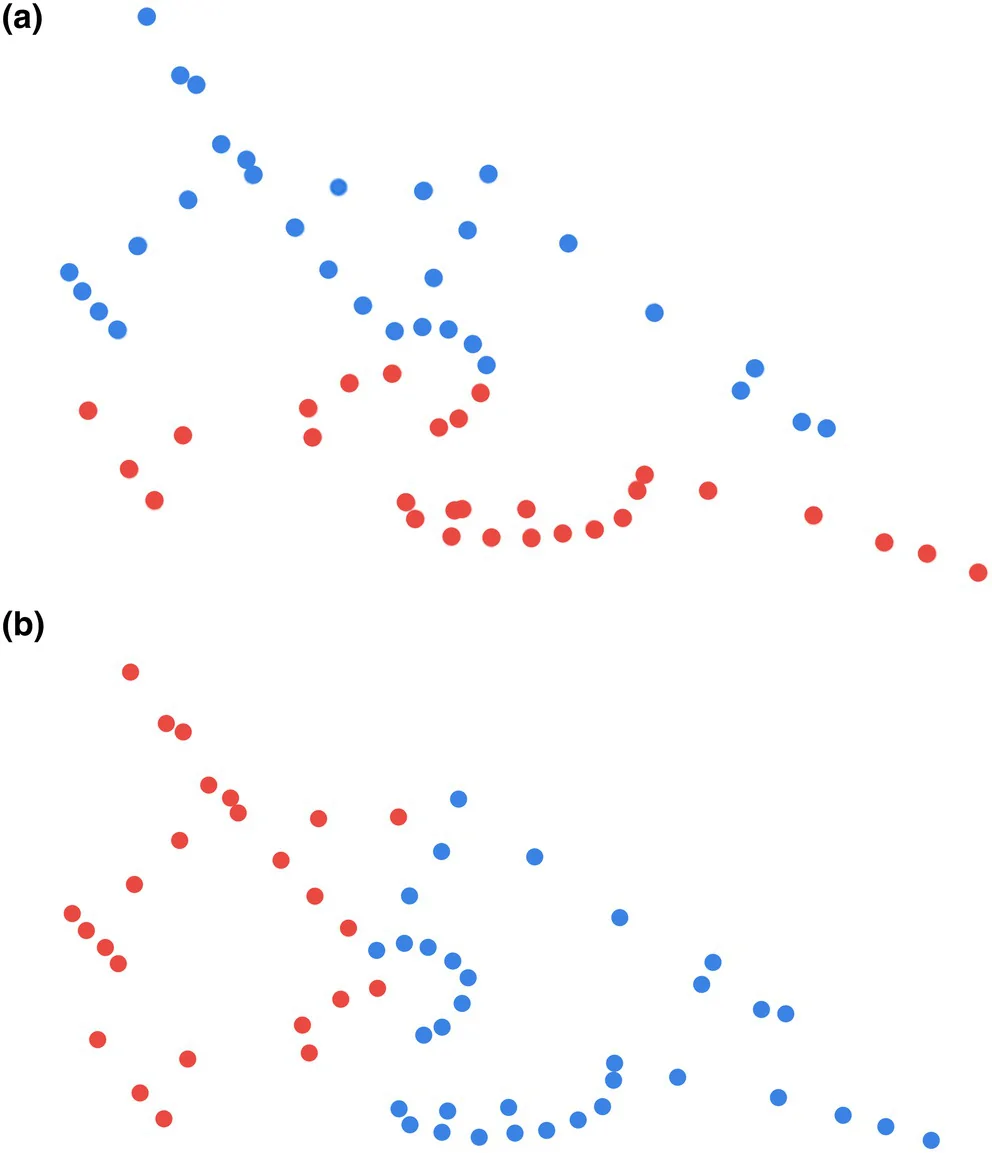
The division of the landmark protocol into two segments for the two different pls analyses. (a) Ventral (Block 1) and dorsal (Block 2). (b) Neurocranium (Block 1) and face (Block 2). The figure represents a *Giraffa* spp. skull in lateral (right) view.

## 3 RESULTS

### 3.1 Taxonomic signal

Concerning females, the principal component analysis (PCA: Figure 3a,b) showed evidence of species‐ and subspecies‐specific discrimination. With a Levene *p* = 0.84, there was a significant difference (*p* < 0.01) between the two South species (*tippelskirchi* and *giraffa*) and the same was seen in the North (*camelopardalis* and *reticulata*) with a Levene *p* = 0.76. The comparisons of subspecies were more specific with Levene *p* = 0.58 for the South and *p* = 0.51 for the North. In these comparisons, the differences between the taxa were less clear with the only significant difference in the South being between the nominate subspecies (*giraffa* and *tippelskirchi*), whereas in the North there were differences between *antiquorum*‐*camelopardalis*, *antiquorum*‐*reticulata*, and *camelopardalis*‐*reticulata*. Main shape differences concerned the height and orientation of the parietal ossicones, the height of the median ossicone, the length of the rostrum, and the width of the maxilla. Interestingly, while the rest of the differences are not statistically significant (*p* > 0.05) for the North group, the *p*‐values are always relatively low (<0.16), showing a faint presence of a signal.

**FIGURE 3:**
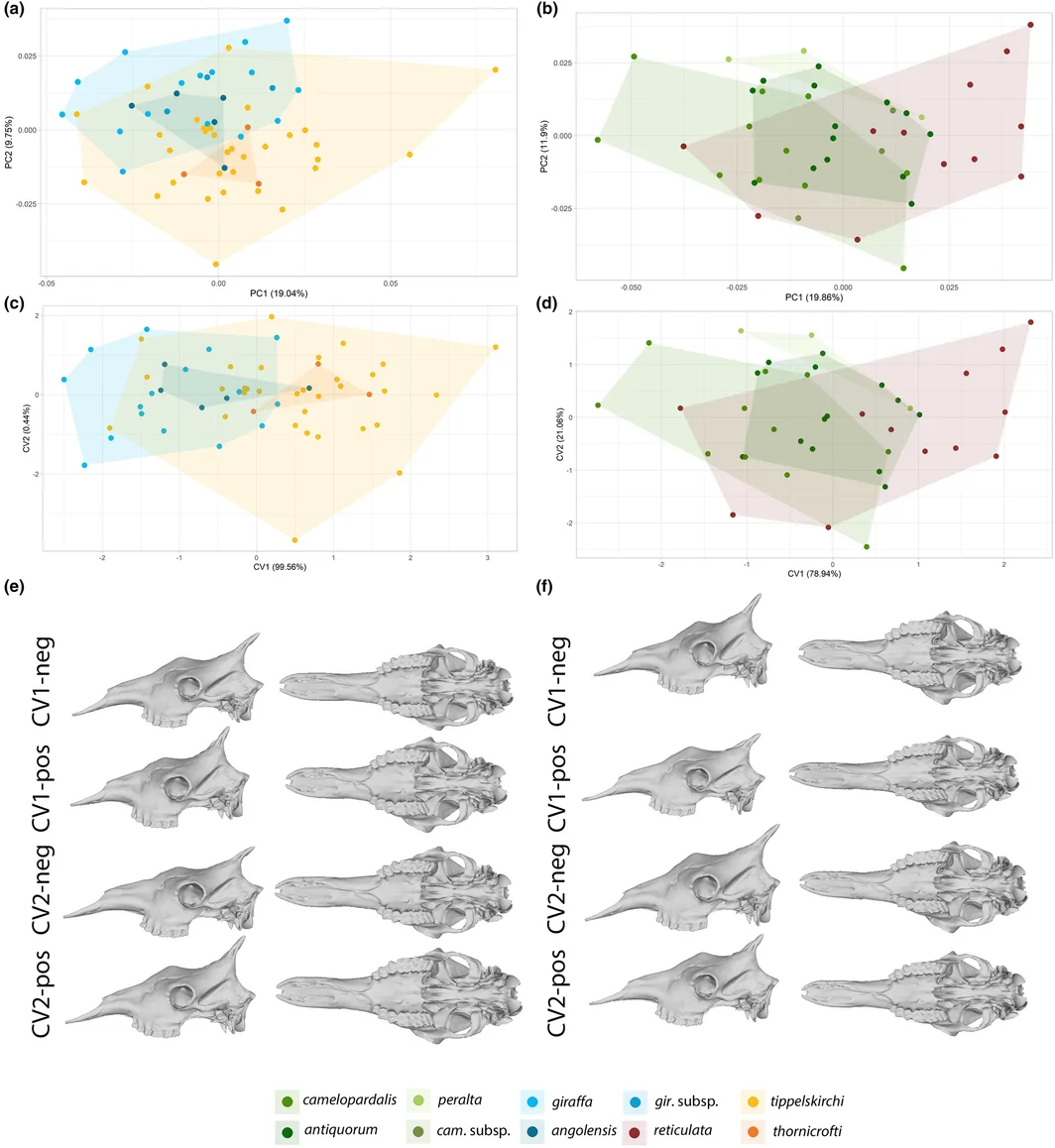
Multivariate analyses on the symmetric component of females of the south (a, c, e) and north (b, d, f) clades using PCA (a, b), CVA (c, d), and phylo‐CVA (e, f). (a) PCA of the south females. (b) PCA of the north females. (c) CVA of the south females. (d) CVA of the north females. (e) Shape changes for PC1 and PC2 for the south females in lateral and ventral view. (f) Shape changes for PC1 and PC2 for the north females in lateral and ventral view. Colour palette: Light blue – *G*. *g*. *giraffa*, medium blue – *G*. *giraffa* subsp., dark blue – *G*. *g*. *angolensis*, yellow – *G*. *t*. *tippelskirchi*, orange – *G*. *t*. *thornicrofti*, dark green – *G*. *c*. *antiquorum*, medium green – *G*. *camelopardalis* subsp., light green – *G*. *c*. *camelopardalis*, khaki – *G*. *c*. *peralta*, and red – *G*. *r*. *reticulata*.

The canonical variate analysis (CVA) Figure 3c,d captured a similar taxonomic signal that is consistent with the phylogeny (Figure 3e,f). The robustness proxies showed mixed results with Levene being always positive (*p* > 0.2), Box M being positive only for the South subspecies, LOO accuracy being positive only for the South group, the CV1 median IQR being positive only for the subspecies. Therefore, even though there is some notable signal in these plots it should be considered as supplementary. Both comparisons of species showed significant differences with *p* < 0.01 between *giraffa*‐*tippelskirchi* and *camelopardalis*‐*reticulata*. Again, the comparisons of subspecies are less clear. Apart from the statistically significant difference between *giraffa*‐*tippelskirchi*, CVAs detect a difference between *giraffa*‐*thornicrofti*. In the North group, the only differences detected are between *antiquorum*‐*camelopardalis* and *camelopardalis*‐*reticulata*. As in the PCA, while the rest of the differences in the North group are not statistically significant (*p* > 0.05), the *p*‐values are always relatively low (<0.13) showing a faint presence of a signal.

Similar to the females, the PCAs of the males (Figure 4a,b) based on the symmetric component of variation only showed evidence of taxonomic signal. Levene *p* was always positive, with the exception of the North species. The results in the PCAs are almost always differentiating between the compared groups, that is, *giraffa*‐*tippelskirchi* and *camelopardalis*‐*reticulata*, all the subspecies of the South clade (*angolensis*, *giraffa*, *thornicrofti*, and *tippelskirchi*) and most of the male subspecies. The only cases where there were no significant differences in the North subspecies were between *reticulata*‐*antiquorum* and *reticulata*‐*peralta*, but in both cases the *p*‐value was low (0.08). As with the females, the main differences in shape concern the size and orientation of the parietal ossicones, the height of the median ossicone, the length of the rostrum, the width of the maxilla and the height of the occiput.

**FIGURE 4:**
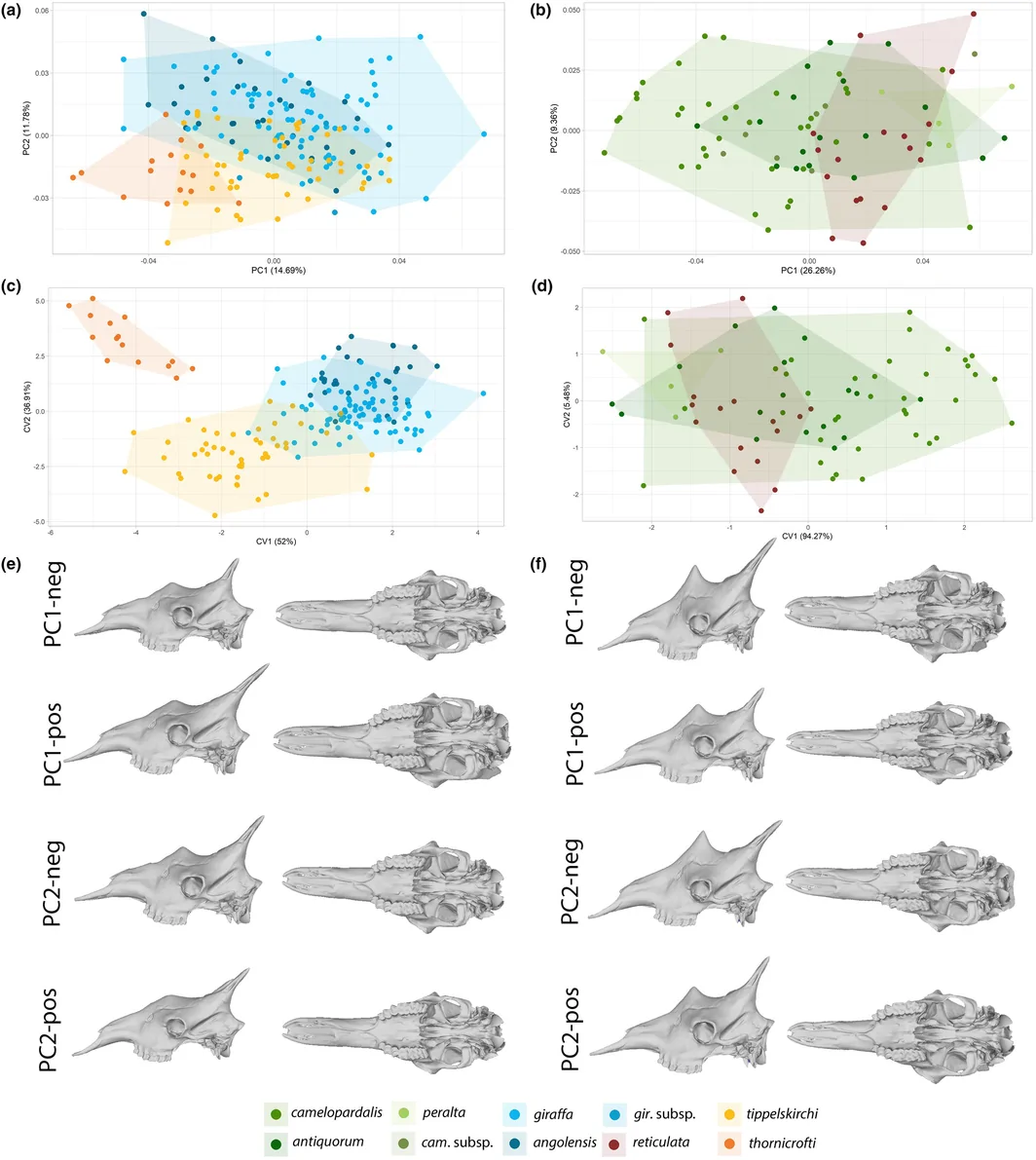
Multivariate analyses on the symmetric component of males of the south (a, c, e) and north (b, d, f) clades using PCA (a, b), CVA (c, d), and phylo‐CVA (e, f). (a) PCA of the south males. (b) PCA of the north females. (c) CVA of the south males. (d) CVA of the north males. (e) Shape changes for PC1 and PC2 for the south males in lateral and ventral view. (f) Shape changes for PC1 and PC2 for the north males in lateral and ventral view. Colour palette: Light blue – *G*. *g*. *giraffa*, medium blue – *G*. *giraffa* subsp., dark blue – *G*. *g*. *angolensis*, yellow – *G*. *t*. *tippelskirchi*, dark green – *G*. *c*. *antiquorum*, medium green – *G*. *camelopardalis* subsp., light green – *G*. *c*. *camelopardalis*, khaki – *G*. *c*. *peralta*, and red – *G*. *r*. *reticulata*.

The CVAs (Figure 4c,d) showed the same statistical differences between the compared groups, following the results of the PCA in every pairwise value, hence corroborating the structure of the data. Again, the two pairs of the north group that did not show statistical significance had a very low *p*‐value (0.11 and 0.08 respectively). Levene *p* was positive for all comparisons, LOO accuracy was negative only for the North subspecies, CV1 median IQR was positive only for the subspecies, whereas Box M *p* was positive only for the North subspecies. Therefore, a combination of positive and negative robustness tests was observed for each analysis and the results are only considered as supplementary to the PCA.

### 3.2 Allometry/ontogeny

The regression between the symmetric component and log centroid size (allometry) of all specimens of known age is shown in Figure 5a. The smallest specimen was a male juvenile zoo specimen that was only 1 week old (ZSM‐1980‐0255, *Giraffa* spp., possible hybrid between *G*. *reticulata* and *G*. *tippelskirchi*), opposed to the largest skull within the dataset from an adult male originating from Namibia (NAM‐TAU‐1606, *G*. *g*. *angolensis*, age class E). Skull shape changes occur uniformly as they grow, and the growth implies clinal differences in the skull, including the face and the ornaments. Statistically significant differences between the centroid sizes of many of the qualitative age classes were detected. The A and B age classes (juveniles) were distinct from every other class and from each other. The same was also true for age classes C and D (subadults), but the difference between them was not significant (*p* = 0.1435). The age classes E–I (adults) showed no significant differences between them (with the sole exception being the pair E and G), but were distinct from the age classes A–D. It can also be noted that the age class B exhibited the widest range of skull size. The total *R* ^2^ was 0.14, indicating small influence of size in variability the sample.

**FIGURE 5:**
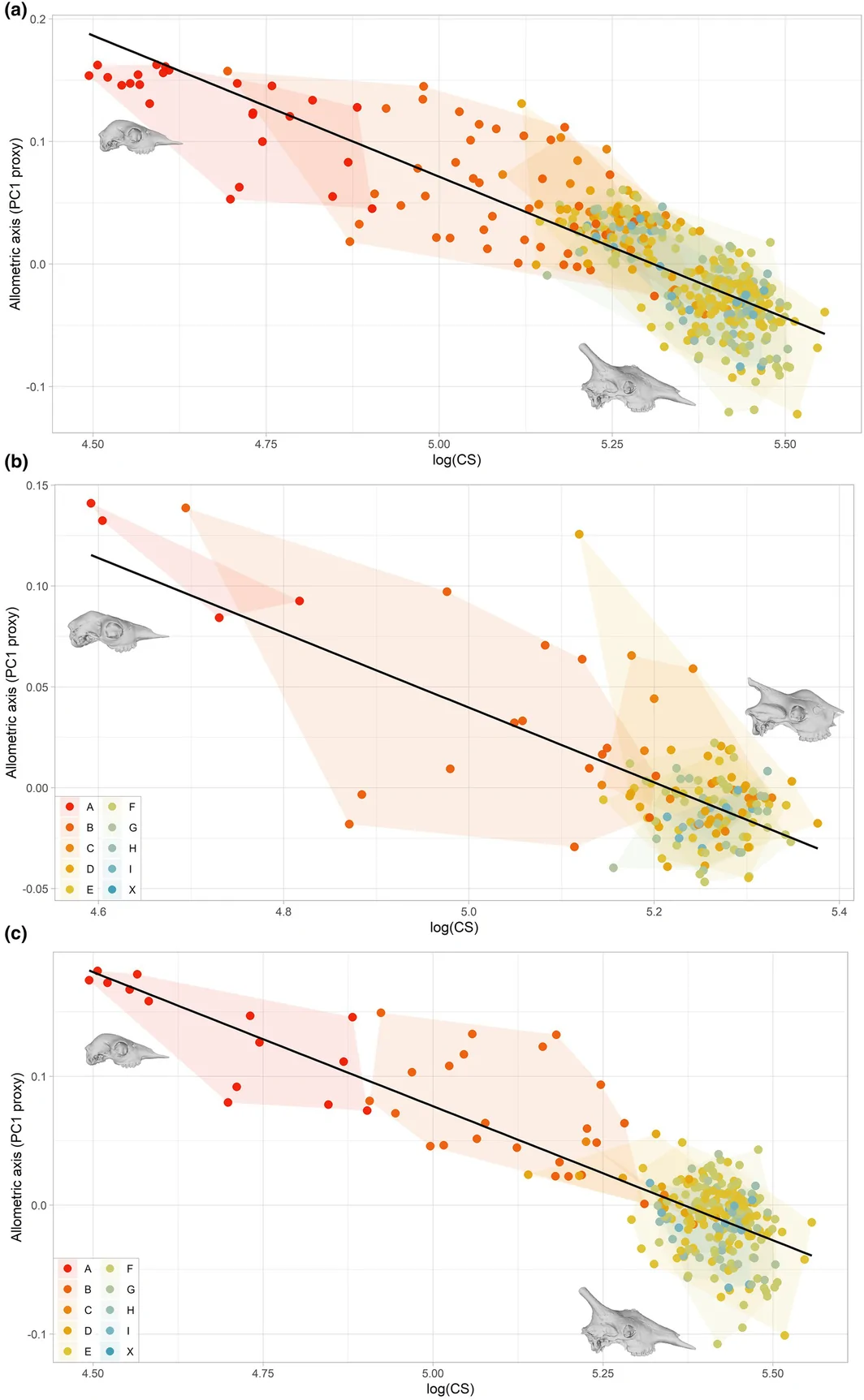
Regression between the symmetric component and centroid size for *Giraffa* specimens of known age classes (A–I). (a) Males and females. (b) Females only. (c) Males only.

Pairwise comparisons between the two sexes and the North/South groups using procD.lm showed statistically significant differences between both the slope and the shift of their distribution. Therefore, they follow distinct ontogenetic trajectories (slope) and have different mean shapes even for similar sizes (slope offset). These comparisons also showed statistically significant differences between the shift of all four species, but non‐significant differences in the slope between *giraffa*‐*reticulata* and *tippelskirchi*‐*reticulata*. Similarly, when subspecies were tested, there were significant differences in the shifts between almost all subspecies (the only exception was peralta‐reticulata) and in most of the slopes (exceptions were *peralta* with everything else except *giraffa*, *camelopardalis*‐*thornicrofti*, and *reticulata*‐*tippelskirchi*). However, phylogenetic generalized least squares analyses (procD.pgls) at the species and subspecies level revealed no significant evolutionary allometry of skull shape with size, nor significant differences in mean skull shape after accounting for size. This indicates that subspecies‐level variation in cranial morphology reflects phylogenetic conservatism rather than independent evolutionary divergence.

Among females, the smallest specimen based on centroid size was SMNS‐Z‐MAM‐7592 (*Giraffa* spp., age class A, Zoo Karlsruhe, 8 days old, possible *G*. *t*. *tippelskirchi*) and the largest specimen was DNMNH‐AZ‐121 (*Giraffa* spp., age class D, Zoo Pretoria) (Figure 5b). Again, there was a clear pattern in their distribution in the plot. In this case, as was the case with the complete dataset, juveniles (age classes A and B) were significantly different from all other classes and different to each other. However, in females, the subadult group (age classes C and D) was not significantly different from the adults with the sole exception being the pair C and F. Therefore, the only significant differences were traced between the juveniles and the others. Again, age class B showed the widest centroid size range. The *R* ^2^ was 0.064 showing little influence of size in the dataset.

When only the males were plotted (Figure 5c), the minimum and maximum sizes were represented by the specimens mentioned for the whole dataset and the results were similar. The age classes A and B were different from every other class (and from each other), and the age classes C and D were different from every other class (but similar to each other). Therefore, a clear differentiation between subadult and adult males is observed, but not so for females. The *R* ^2^ now was 0.11. LOO accuracy was strong for all three analyses shown in Figure 5.

To remove the ontogenetic influence of the juveniles and subadults in the study of allometry, only adults (age classes E–I) were plotted (Figure 6). When males and females were plotted together (Figure 6a), there was a relatively clear distinction between the two in the distribution of the specimens. Pairwise comparisons showed statistical differences only between the age classes E and G. No statistical differences between any of the adult age classes were found in the separate analyses for the females (Figure 6b) and males (Figure 6c). In these three analyses, the *p*‐value was always <0.01 showing that there is evidence of static allometry in the adults, even if it is not correlated to age classes. The LOO accuracy was strong for the complete dataset and for the males, but weak for the females (0.6893).

**FIGURE 6:**
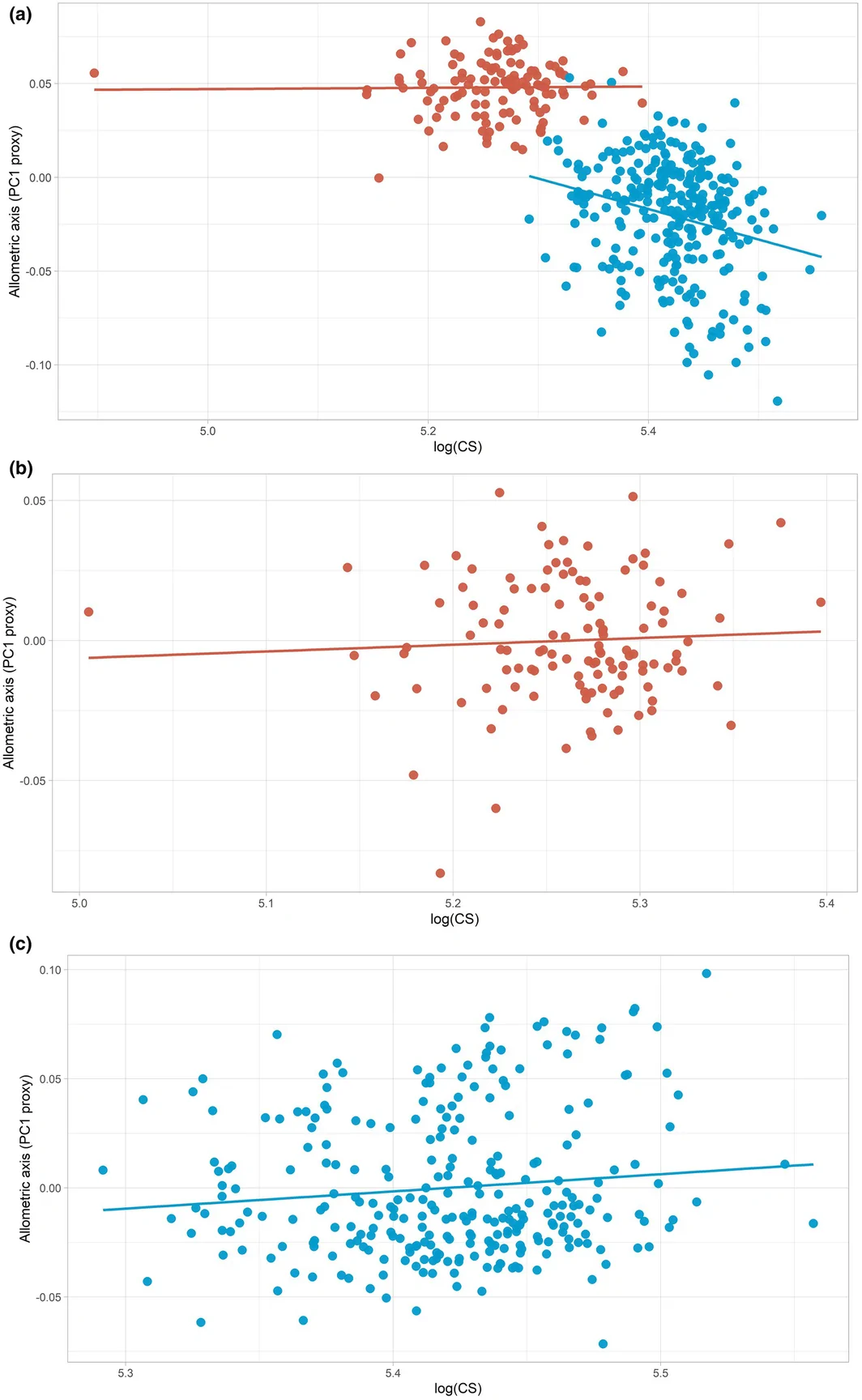
Regression between the symmetric component and the loc centroid size for adult *Giraffa* specimens of age classes (E–I). (a) Males and females. (b) Females only. (c) Males only.

### 3.3 Integration/modularity

The 2B PLS between the dorsal and the ventral parts of the skulls showed that the two blocks covariate significantly (*r* = 0.9406; Figure 7a). For the face‐neurocranial comparison (Figure 7b), a very similar result was found, with significant covariation (*r* = 0.83). There was a marked differentiation between the two sexes, and the shape difference across the axes was striking, concerning the ossicones, the orbits, the palate and the occiput. For both cases, the 2BPLS Effect Size (Z) was positive (5.83257 and 5.65016, respectively) and the 2B PLS *p*‐values were <0.001, verifying that there is very strong covariation between the compared blocks. However, the modularity tests showed that there is clear modularity between the compared blocks, with *p*‐value <0.001. The modularity effect size was negative with −4.03 for the ventral‐dorsal analysis and −6.42 for the neurocranium‐face analysis. Therefore, even though there is strong covariation of the compared cranial parts, there seems to be a certain degree of modularity in the variability.

**FIGURE 7:**
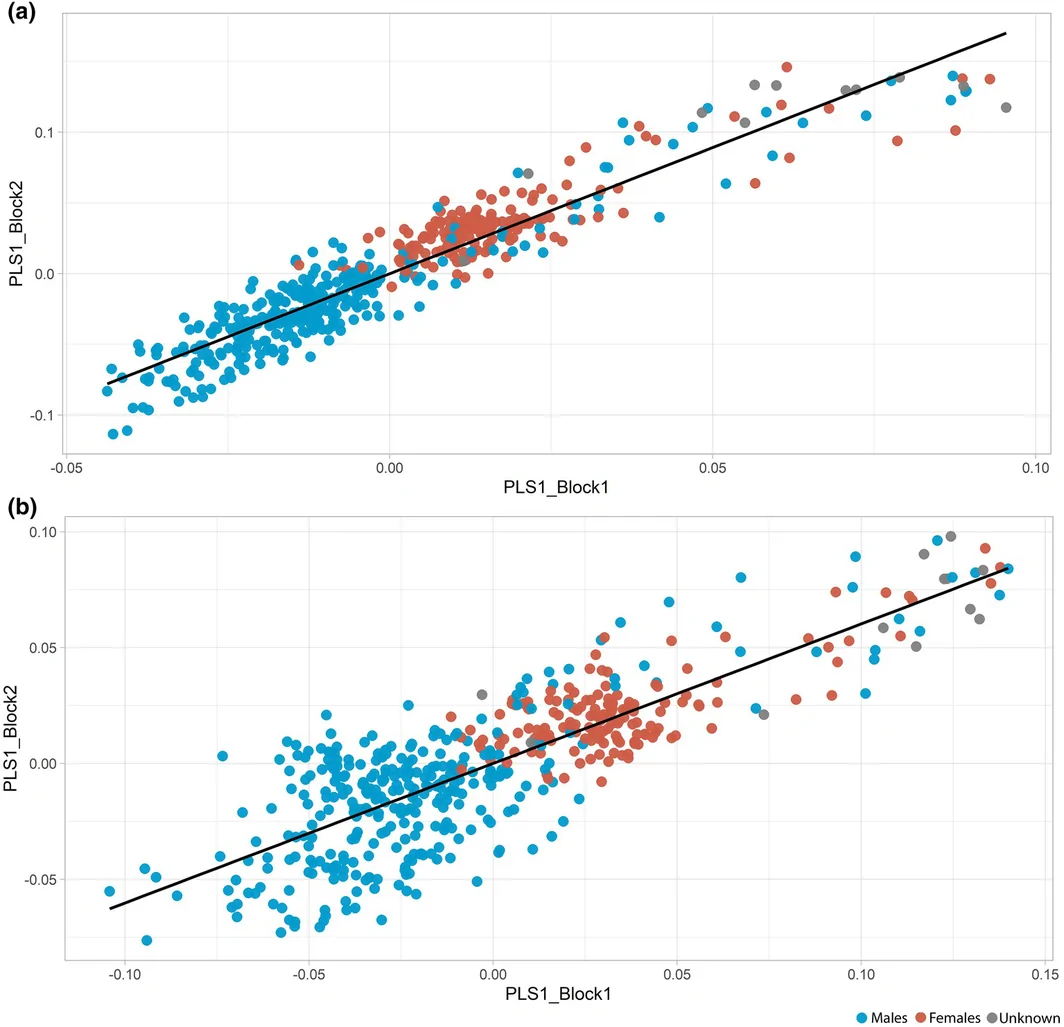
2B PLS for the symmetric component. (a) Ventral (Block 1) and dorsal (Block 2). (b) Neurocranium (Block 1) and face (Block 2).

## 4 DISCUSSION

The geometric morphometric analysis of extant *Giraffa* skulls revealed how its morphological variation is shaped by developmental constraints, sexual dimorphism, and biogeographic structure – ultimately tying into social and communicative functions. Here, we discuss our results in terms of (i) taxonomic and evolutionary implications, and (ii) allometry/ontogeny and integration/modularity.

### 4.1 Taxonomic and evolutionary implications

Until recently, the taxonomic status of the distinct extant *Giraffa* forms has been a matter of debate, with the IUCN accepting the simplest solution of recognizing only one species of giraffe (*Giraffa camelopardalis*) and nine subspecies, in accordance with the studies of Singer and Boné (1960), Dagg (1962, 1971, 2014), and Seymour (2001). However, a 2025 IUCN evaluation (IUCN SSC GOSG Task Force, 2025) agreed with recent evidence supporting the taxonomic split of the extant *Giraffa* into four distinct species (*G*. *giraffa*, *G*. *camelopardalis*, *G*. *reticulata*, and *G*. *tippelskirchi*), based on genetic, ecological, and osteological data (Brown et al., 2023; Coimbra et al., 2021, 2022, 2023; Fennessy et al., 2016; Kargopoulos et al., 2024; Lee & Solounias, 2022; Muneza et al., 2025; Petzold et al., 2020; Petzold & Hassanin, 2020; Thomassen et al., 2013; Winter et al., 2018). Taxonomy is pivotal for the conservation of threatened species (Costello et al., 2015; Mace, 2004; Morrison III et al., 2009; Schmidt et al., 2023; Seymour, 2001; Vogel Ely et al., 2017), and *Giraffa* are no exception (Bertola et al., 2024; Coimbra et al., 2021, 2023; Fennessy et al., 2016; Kargopoulos et al., 2024; Muneza et al., 2025; Winter et al., 2018).

Kargopoulos et al. (2024) applied a 3D Geometric Morphometrics approach that statistically supported the taxonomic split of *Giraffa* species. Our more detailed analyses in this study agrees with this conclusion, as more detailed differences between the cranial shapes of each of the species and subspecies were detected. Importantly, the PCA and CVA results reflect evolution matching geography in the same manner, further showing that all skull traits vary concomitantly (face, orbits, and ornaments). In accordance with the results presented in Kargopoulos et al. (2024), and in further development of the latter, it was found that statistically significant differences were found in all performed PCAs and CVAs (Figures 3 and 4). The differences were slightly more evident in the south clade in both females (Figure 3a,c,e) and males (Figure 4a,c,e) in comparison with the respective ones in the north clade (Figures 3b,d,f, 4b,d,f). Consequently, it is verified that the cranial differences between species are indeed valid, further augmenting the argument of distinct cranial morphologies and their consequent taxonomic and conservational impact as presented in Kargopoulos et al. (2024) and Muneza et al. (2025). It is evident that in many cases. geography affects the distribution of the studied groups in the analyses. Moreover, the encompassed differentiating traits vary concomitantly implying visual cues. Furthermore, the results show that in most cases, such shape features differentiate logical species groupings. This was also noted clearly in the procD.lm comparisons of the slopes and shifts of the North/South groups, species, and subspecies. Therefore, a strong influence of phylogeny is present in the current variability shown in cranial shape of the modern *Giraffa*. This is supported by the conservatism revealed by the procD.pgls results that showed no significant independence in the evolutionary divergence of the species or subspecies.

Nevertheless, the interpretation of such morphological differences as definitive taxonomic boundaries of extant *Giraffa* has been subject to debate. Critics (e.g., Solounias, 2007) have questioned the validity of species‐level separation in the absence of functional evidence for resource partitioning, mate recognition, or reproductive barriers. However, such objections often stem from a classical view of speciation that emphasizes reproductive isolation as a necessary condition – an assumption that may not hold in systems characterized by reticulate evolutionary histories. Owen‐Smith (2025) recently expressed whether the differences between the *Giraffa* taxa show ecological or evolutionary significance are applicable in a species definition from a more ecological point of view. However, in light of our results, the role of cranial shape variation in the evolution of *Giraffa* is unambiguously important and reflects its pivotal position as a defining trait in the taxonomic framework of the family. Along with other obvious traits that establish individual or species identity, cranial ornaments are components that can be interpreted as visual cues, designed to trigger specific responses (e.g., Keil et al., 2012). Visual cues as drivers of recognition are ultimately congruent with complex social structures. Furthermore, given the advanced visual capacity of extant *Giraffa* (Coimbra et al., 2013; Ishengoma et al., 2017; Kasozi & Montgomery, 2018; Mitchell et al., 2013) and the variability in the orbit architecture (Kargopoulos et al., 2024), ossicones clearly fit into this category and play an important role in their evolution.

Based on the results, there is a phenotypic logic underlying diversification that is largely geographic and congruent with reticular evolution. The current geographic distributions of the distinct species and subspecies of modern *Giraffa* have been interpreted as evidence of parapatric evolution (Brown et al., 2007; Coimbra et al., 2023; Thomassen et al., 2013). This implies that geographical variations of a species become reproductively isolated over a certain time, for example, Gavrilets et al. (2000), Butlin et al. (2008). Thomassen et al. (2013) proposed that this reproductive isolation was achieved through reproductive asynchrony correlated with regional rainfall patterns in East Africa. Brown et al. (2007) and Bertola et al. (2024) proposed that reproductive isolation could be achieved through temporary allopatry in a mixing‐isolation‐mixing pattern, caused by environmental barriers. In *Giraffa*, multiple genomic studies (e.g., Bertola et al., 2024; Coimbra et al., 2021; Fennessy et al., 2016) document historical and ongoing gene flow between lineages, while still recognizing them as distinct evolutionary units. This suggests that complete reproductive isolation is not a prerequisite for species differentiation in these taxa. Instead, *Giraffa* speciation appears to reflect a dynamic balance between local divergence, genealogical connectivity, and social or ecological adaptation. Notably, our results indicate that morphological features such as ossicone shape may encode consistent individual signatures, functioning as visual identity cues within and potentially between species. These cues likely contribute to social cohesion and mate recognition in a visually dependent, fission – fusion social system. In this framework, morphological variation – including ossicone traits – should not be interpreted as strict markers of isolation, but rather as components of a complex evolutionary landscape in which phenotypic identity, structural integration, and evolutionary history interact in non‐linear and functionally meaningful ways.

An additional way in which parapatric or sympatric reproductive isolation takes place is through sexual selection, meaning that certain populations of a species develop distinct mating behaviours or even distinct secondary sexual characters that affect their reproductive success (Cooney et al., 2017; van Doorn et al., 2004). Concerning modern *Giraffa*, such sexual characters could include a range of anatomical differences, since they are highly sexually dimorphic (Dagg, 1968, 1971, 2014; Seymour, 2001; Shorrocks, 2016). Since this study focused on their cranial anatomy, the sexual characteristic considered herein as possibly related to sexual selection is the shape of the head, mostly affected by the shape of the ossicones.

Intersexual and intrasexual interactions related to reproduction are very important to understand the behavioural framework of sexual isolation. There have been several behavioural observations related to reproduction (e.g., Krumbiegel, 1971; Pratt & Anderson, 1985), and they could potentially aid in interpreting the cranial morphological variability shown here. In particular, the observations of Pratt and Anderson (1985) are of special interest, since they reveal important patterns. In their study, male *G*. *t*. *tippelskirchi* from Tanzania were grouped in three classes (A, B, and C) based on their robustness and the degree of development of their ossicones or other minor ossifications of the skull. They found that males belonging to the most robust class (A) never engaged in intrasexual fighting, because their intimidating presence was enough to make them dominate a territory or a herd, since other males (of class B or C) avoided clashing with them. Additionally, a key‐point of extant *Giraffa* reproduction is urine‐testing, in which males urge females to urinate so that they can test if they are in oestrus. However, females do not respond randomly to this behaviour, since they may deny urinating for males that are visually not meeting a certain standard (Dagg, 2014; Pratt & Anderson, 1985).

We propose that the shape of the skull and the contribution of the ossicones are important for the evolution of the genus *Giraffa*. Sexual isolation leading to parapatric speciation could have been caused by environmental factors (Bertola et al., 2024; Brown et al., 2007), reproductive asynchrony (Thomassen et al., 2013), and/or based on cranial morphology, which affects the reproductive success of males through their interactions with females and with other competing males. Judging from the aforementioned behavioural observations, males with larger ossicones and more distinct cranial ossifications (median ossicone, nuchal crest, and additional ossifications on the dorsal profile of the skull) are more dominant and hence more reproductively successful.

Several fossil species of *Giraffa* have been found across Africa and a few in Asia (e.g., Geraads et al., 2013; Hamilton, 1978; Harris, 1976a, 1976b; Harris et al., 2010; Mitchell & Skinner, 2003; Shorrocks, 2016; Singer & Boné, 1960). However, there is some disagreement concerning the possible ancestor of modern *Giraffa*, as Hamilton (1978) suggests that it is *Giraffa jumae*, whereas Harris (1976a), Mitchell and Skinner (2003), and Geraads et al. (2013) suggest that it is *Giraffa gracilis*. In any case, both forms have less developed cranial appendages than extant *Giraffa*, especially regarding the median ossicone and the development of the additional ossifications (Harris et al., 2010, and references therein). In this sense, the morphology seen in the extant *Giraffa giraffa* seems closer to the plesiomorphic characteristics of these lineages, whereas the more developed median ossicone, nuchal crest, and the additional ossifications seen in other species are derived characters.

Given that our knowledge of the cranial anatomy of the fossil species is limited, such conclusions are uncertain but are reasonable interpretations based on the morphological diversity of *Giraffa*. However, if this evolutionary ordination is followed, it conforms to the behavioural observations mentioned before. Since males with more robust or more numerous cranial appendages have higher reproductive success (Dagg, 2014; Pratt & Anderson, 1985), it is reasonable to suggest that these forms are derived as compared with ones that exhibit the plesiomorphic features with more simplified morphologies.

An intriguing question is whether the more ancestral‐like morphology of *G*. *giraffa* is correlated with ancestral traits (plesiomorphy) or is perhaps evidence of heterochrony (paedomorphosis), since smaller cranial appendages are also typical of juveniles (Fink, 1982; Kargopoulos et al., 2024; Vinicius & Mirazon Lahr, 2003). Similar uncertainties exist for other taxa, for example, antelopes (Vrba et al., 1994), marsupials (White et al., 2023), and rodents (von Koenigswald, 1993). However, among *Giraffa*, the relative phylogenetic isolation poses a challenge, since the only other extant genus of the family is the poorly represented okapi, along with the fact that fossil giraffids are poorly known.

### 4.2 Allometry/ontogeny – integration/modularity

Regressions of skull shape to skull size follow the same trend for both sexes and for all extant *Giraffa* species. As briefly mentioned in Kargopoulos et al. (2024) this shows that they follow a conservative developmental programme regardless of their complex taxonomy. Sexual dimorphism is evident developmentally because males grow larger, changing further along the same allometric direction. Static allometry for adults is negligible, and in the cases of larger females, their morphology tends to resemble that of males. However, as indicated by the procD.lm comparisons, there are statistically significant differences in the slope and shift between males and females, showing an underlying divergence in the ontogenetic trajectories, which differ in direction and also show that males and females of the same centroid size have at any moment different cranial shapes.

According to the obtained growth trajectories (Figure 5), there are three stages in modern *Giraffa* ontogeny that match qualitative features of skull morphology; the first stage (juveniles) includes the age classes A and B and corresponds to animals of 0–4 years of age. Since all specimens follow a relatively homogenous ontogenetic trajectory, accordingly, the males do not stop their cranial growth during the subadult age class but continue growing for another 4 years until they reach their final size by the age of 8 years, corresponding to age class E, which coincides with their age of reproductive maturity (Berry & Bercovitch, 2012; Dagg, 2014). In contrast, female skulls seem to stop growing at age class C, around 4 years old, which is the approximate age at which they become reproductively mature (Bercovitch & Berry, 2013; Dagg, 2014). This growth pattern aligns with assessments on body weight and total height respectively (Dagg & Foster, 1982; Hirst, 1975) as well as with ages of reproductive maturity (Bercovitch & Berry, 2013; Berry & Bercovitch, 2012). Additionally, age class B (2–4 years old) shows the widest range of variation in skull size, entailing that during this period, cranial size increases more drastically, coinciding with the age in which the calves have stopped weaning and are fully independent in terms of feeding (Dagg, 2014). Despite the high content in fat and protein, at least in comparison to bovine milk (Dagg, 2014; Dagg & Foster, 1982; Petzinger et al., 2013; Shorrocks, 2016), it might be the dietary independence of the calves, which provides them enough nutrients to lead to this surge in cranial development. In males, the development seems to be more gradual, with similarities within the groups A‐B‐C and B‐C‐D, in comparison to the clearer pattern A‐B and C‐D shown in the complete dataset. The interesting similarities between the age classes C, H, and I for males can be explained by the gradual wear of the ossicones in very old animals.

A common allometric trajectory explains about 15% of the shape variation across all species and sexes, involving coordinated craniofacial changes, where the skull changes with size, while the ossicones grow disproportionately, that is, hence differentiating males. Interestingly, the allometric trajectory itself may be interpreted as a first level of integration – a developmental rule by which size governs coordinated shape change across multiple cranial regions (Klingenberg, 2016) (discussed in more detail below). This covariation is not random but reflects underlying biological constraints that canalize growth along specific pathways. In extant *Giraffa*, this pattern is shared across sexes and species, suggesting a deeply conserved integration axis (Cavener et al., 2024).

Analysis of modularity showed an unexpected combination of results. There is clear evidence of strong covariation of the studied cranial parts through the 2BPLS with high Z and *p* < 0.001, but at the same time, there is clear effect of modularity with negative Effect Size (*p* < 0.001). Therefore, we notice that there is some degree of modularity in the magnitude of the changes that occur in the cranium (i.e., more drastic changes in the ossicones), but at the same time, there is a covariation of the discussed parts along the PLS axes, showing a linkage in the developmental trajectory. This covariation between modules, such as the craniofacial region and ossicones, suggests that to some degree, despite the magnitude of changes of the ossicones, the skull functions as a cohesive morphological unit (Goswami & Polly, 2010). This likely reflects the multifunctionality of the *Giraffa* skull: it must simultaneously support feeding biomechanics, sexual combat, sensory perception, and visual signalling. High integration constrains local morphological flexibility but ensures structural coordination across multiple ecological and behavioural demands (Lieberman, 2011). Furthermore, this can be interpreted as a reflection of deep developmental constraints, where the units of the skull are so tightly coordinated that independent variation becomes limited (Klingenberg, 2008). Because such constraints reduce the degrees of freedom available for local adaptation or rapid morphological innovation (Klingenberg, 2008; Wagner, 1988), in *Giraffa* this probably explains why features directly tied to the cranial architecture, such as facial proportions or orbital positioning, remain relatively stable across taxa.

Many mammalian taxa with cranial ornaments have shown modularity in cranial ornaments (e.g., wildebeest Gerstenhaber & Knapp, 2022), but cranial integration can differ drastically even between closely related ruminant taxa (Haber, 2015). Therefore, the combination of modularity and covariation noticed in modern *Giraffa* is unexpected and should be further investigated, with a focus on the developmental alignment of ossicones with facial physiognomy, for example, orbits. However, this is not the first time that *Giraffa* growth has shown unexpected patterns, since unique developmental mechanisms were reported in the neck (Colalillo & Solounias, 2023; Solounias, 1999), the ossicones (e.g., Spinage, 1968), and in limb bone dimensions (McMahon, 1975) and osteohistology (Smith, 2020), as well as associated with their unusual genetic history (Hudson, 2019). As the overall morphology of modern *Giraffa* is rather strikingly unique among modern mammals, it appears fitting that the developmental programmes that govern its growth ought to be exceptional. Of course, the highly variable environment has played a significant role in their evolution and may explain features as important as the observed variation that makes most giraffe look alike, for example, phenotypic plasticity. However, in *Giraffa* skulls, morphological integration appears consistent across species despite environmental (geographic) variation. This consistency suggests that integration is primarily under genetic control, as environmental differences would likely produce greater variability in integration patterns. Thus, skull integration can be hypothesized to be genetic and linked to its role in visual recognition, tied to their visual acuity, which is also inherited.

Importantly, however, our analyses show that even structures like the ossicones covary with the rest of the skull. This suggests that they do not evolve completely independently, but as part of a coordinated cranial complex. Interestingly, components of skull variation that include asymmetry also covary across individuals, which points to the presence of structured, system‐wide developmental and/or functional influences. Rather than reflecting random noise (Palmer & Strobeck, 1986; Van Valen, 1962), this patterned asymmetry may carry consistent individual signatures, potentially usable as visual identity cues (Parr & de Waal, 1999). In such a scenario, even within an integrated framework, the system retains enough flexibility to encode socially relevant phenotypic variation in predictable ways. Notwithstanding this, although the ossicones are structurally integrated into the cranial module, they appear to exhibit greater lability than other areas. Rather than being fully independent, they may represent a flexible module whose variability is permitted within the limits of a globally integrated system – this is consistent with frameworks where modularity and integration are not mutually exclusive but operate simultaneously (Burns et al., 2023). The coexistence of modularity and integration can promote rapid and extensive morphological diversification, as it enables structured yet flexible variation throughout the organism, allowing the modification of overall features of shape variation without losing functional coordination (Burns et al., 2023). In this context, it becomes plausible that other visible traits – such as pelage patterns – may have evolved as compensatory identity signals within a constrained cranial system. Modern *Giraffa* coat patterns are highly individual and heritable (Lee et al., 2018) and may serve as evolutionarily flexible ‘relief valves’ for social recognition, especially given the species' reliance on vision and its dynamic fission–fusion social structure. These traits, along with ossicone morphology, likely provide modular avenues for adaptive variation in identity without compromising cranial integration. In such visually oriented species, the head remains a critical focal point for individual recognition, particularly through ossicone morphology and overall cranial shape. Even under strong integration, the giraffe's head can be seen functioning as a stable yet informative social signal, complementing the pelage as part of a multimodal visual system for identity and cohesion.

Modern *Giraffa* inhabit ecologically diverse and variable landscapes, where selective pressures fluctuate across both space and time. Theoretical models suggest that in such environments, morphological modularity enhances evolvability by allowing localized adaptation without compromising whole‐organism functionality (Neaux, 2017; Wagner et al., 2007). However, they display a paradox: they retain a highly integrated skull despite living in unstable environments. One explanation may be that selection has not relaxed integration broadly but has instead channelled adaptive flexibility into discrete visual traits, such as ossicones and pelage patterns. These traits act as externally modular systems, capable of encoding individual, sex, or species identity, while the core cranial framework remains evolutionarily conserved.

## 5 CONCLUSIONS

Female giraffe complete cranial growth earlier in age compared with males, corresponding to the attainment of sexual maturity for each sex, while static allometry is present. The skull of the extant *Giraffa* shows a combination of modularity and modular covariation, showing a homogenous evolutionary and developmental pattern. Significant differences were found in the PCAs and CVAs between the four species as well as between subspecies of the same species. The ossicones have been pivotal to the evolution of *Giraffa*, acting in sexual selection that drives parapatric evolution. Overall, our findings have broader implications for macroevolution and conservation. Morphological covariation, while advantageous for functional coherence, may limit the phenotypic diversity needed for adaptation under rapid environmental change, thus increasing extinction risk. In contrast, species with modular traits may better navigate environmental uncertainty. The evolutionary strategy of *Giraffa –* combining internal constraint with external flexibility – offers a striking example of how identity, function, and evolution intersect in large vertebrates, and underscores the value of integrative approaches in evolutionary biology and conservation planning.

## AUTHOR CONTRIBUTIONS

Conceptualization: Nikolaos Kargopoulos, Jesús Marugán‐Lobón, Anusuya Chinsamy‐Turan, Stephanie Fennessy, Julian Fennessy. Data acquisition: Nikolaos Kargopoulos, Bernard R. Agwanda, Michael Butler Brown, Sara Ferguson, Rigardt Hoffman, Fredrick Lala, Arthur Muneza, Ogeto Mwebi, Moses Otiende, Alice Petzold, Sven Winter, Abdoul Razack Moussa Zabeirou. Formal analysis: Nikolaos Kargopoulos, Jesús Marugán‐Lobón. Funding acquisition: Stephanie Fennessy, Julian Fennessy. Investigation: Nikolaos Kargopoulos, Jesús Marugán‐Lobón, Anusuya Chinsamy‐Turan, Stephanie Fennessy, Julian Fennessy. Supervision: Jesús Marugán‐Lobón, Anusuya Chinsamy‐Turan, Stephanie Fennessy, Julian Fennessy. Visualization: Nikolaos Kargopoulos. Writing‐original draft preparation: Nikolaos Kargopoulos, Jesús Marugán‐Lobón, Anusuya Chinsamy‐Turan, Stephanie Fennessy, Julian Fennessy. Writing‐review and editing: Nikolaos Kargopoulos, Jesús Marugán‐Lobón, Anusuya Chinsamy‐Turan, Stephanie Fennessy, Julian Fennessy.

## ETHICS STATEMENT

No ethical requirements for research animals in this study were needed, as the main surveyed specimen consists of cranial material. No animal was specifically sacrificed for this study.

## Supporting information


Figure S1.

Figure S2.

Figure S3.

Figure S4.

Figure S5.

Table S1.

## Data Availability

The code can be found on https://github.com/nkargopoulos/Giraffe‐Cranial‐3D‐GM. The 3D scans of all the giraffe skulls will be uploaded to Morphosource upon communication with the hosting collections.
